# Supplementary material for: MetaRibo-Seq measures translation in microbiomes
Source: Nat Commun. 2020 Jun 29;11:3268. doi: 10.1038/s41467-020-17081-z (PMC7324362; doi:10.1038/s41467-020-17081-z)
Supplement: Supplementary file 10 — Supplementary Data 7 [file 41467_2020_17081_MOESM10_ESM.zip › File2/Confidence_High_Taxonomy/99_out.krona.html]

Javascript must be enabled to view this page.

members
magnitude
magnitudeUnassigned
count
unassigned
taxon
rank

99\_out

15

15
superkingdom
2

phylum
976
15

200643
class
15

order
171549
15

2
family
1853231

574697
genus
1

1

SRS020394\_contig\_number\_15569
species
544645

1
genus
283168


SRS019068\_contig\_number\_93987
28118
species
1

815
family
12

12
816
genus

1

SRS893378\_contig\_number\_367
species
818

46506
species

SRS011084\_contig\_number\_33643SRS011302\_contig\_number\_20877SRS015431\_contig\_number\_31562SRS024331\_contig\_number\_contig-100\_2302.174097SRS045004\_contig\_number\_23843SRS046369\_contig\_number\_440SRS049896\_contig\_number\_19776SRS077589\_contig\_number\_8036SRS1041147\_contig\_number\_530SRS147557\_contig\_number\_21229SRS149181\_contig\_number\_10966
11

1
171552
family

838
genus
1


SRS143070\_contig\_number\_2969
1262930
species
1
